# Supplementary material for: Discerning Developmental Dyscalculia and Neurodevelopmental Models of Numerical Cognition in a Disadvantaged Educational Context
Source: Brain Sci. 2022 May 16;12(5):653. doi: 10.3390/brainsci12050653 (PMC9139865; doi:10.3390/brainsci12050653)
Supplement: Supplementary file 1 [file brainsci-12-00653-s001.zip › brainsci-1594211-supplementary.pdf]

Supplementary Materials

Table S1 – Performance on verbal QI, RCPM, and SAT total tests in Brazilian children per gender

	Girls	Boys	t	p
Verbal QI – WISC III (*n*= 134)*	104.16 (11.58)	106.77 (12.72)	-1.24	0.22
RCPM (*n*= 169)**	67.17 (19.28)	67.00 (22.10)	0.05	0.96
School Achievement Test – SAT (*n*= 118)***				
Written subtest	19.76 (7.23)	20.89 (6.88)	-0.87	0.39
Arithmetic subtest	12.40 (5.14)	11.67 (4.36)	0.84	0.40
Reading subtest	57.53 (10.78)	58.06 (11.15)	-0.26	0.79
Total	89.68 (20.67)	90.41 (19.72)	- 0.19	0.85

Legend: WISC III: Wechsler Intelligence Scale for Children; RCPM: Raven's Coloured Progressive Matrices; SAT: School Achievement Test. *67 boys and 67 girls; **76 girls and 93 boys; ***55 girls and 63 boys

Table S2. Reliability results for a subset of participants for abstract reasoning and numerical cognition measures.

	Pre-test (n=14)	Post-test (n=14)	Wilcoxon Z	Wilcoxon P	Spearman *R*	Spearman p
CPM	61.78 (18.56)	70.71 (20.46)	-2.35[b]	0.02	0.76	0.002
Counting dots[a]	3.36 (0.93)	3.18 (1.88)	-0.58[b]	0.56	0.11	0.71
Counting backwards[b]	2.03 (1.36)	2.80 (1.35)	-1.98[b]	0.04*	0.62	0.02*
Dictation of numbers[c]	6.33 (3.90)	12.18 (3.91)	-1.24	0.21	0.91	<0.001*
Mental calculation[c]	12.19 (9.00)	22.62 (11.37)	-1.74	0.08	0.85	<0.001*
Reading numbers[d]	7.92 (4.63)	13.20 (3.68)	-0.138[c]	0.89	0.86	<0.001*
Memory of Digits	21.34 (5.64)	23.24 (7.02)	-2.39[b]	0.02*	0.70	0.005*
Positioning on a number line[b]	11.13 (5.88)	15.49 (4.74)	-0.55[b]	0.58	0.80	0.001*
Oral comparison[d]	10.92 (3.42)	12.65 (2.81)	-1.41[c]	0.16	0.28	0.33
Perceptual estimation[e]	5.50 (2.36)	6.08 (2.49)	-0.14[c]	0.89	0.18	0.53
Contextual estimation[f]	8.39 (3.99)	10.30 (4.50)	-1.28[b]	0.20	0.56	0.04*
Problem solving[d]	2.81 (3.25)	5.42 (3.58)	-0.57[b]	0.57	0.49	0.07
Written comparison[g]	16.72 (3.03)	18.46 (2.68)	-1.34	0.18	0.87	<0.001*
Zareki-R Total[d]	99.07 (43.15)	134.05 (332.25)	-3.24	0.001*	0.99	<0.001*
Score A	56.89 (20.38)	84.53 (22.22)	-1.61[b]	0.11	0.94	<0.001*

Legend: CPM=Raven's Coloured Progressive Matrices. * < 0.05.

Table S3. Correlation between Zareki-R and Arithmetic of WISC-III or Arithmetic of SAT.

	**Arithmetic of WISC-III (*N*=122)**	**Arithmetic of SAT (*N*=118)**
Counting dots	0.18	0.26
Counting backwards	0.49*	0.45*
Dictation of numbers	0.49*	0.54*
Mental calculation	0.62†	0.70†
Reading numbers	0.49*	0.47*
Positioning on a number line	0.39	0.28
Oral comparison	0.41	0.44*
Perceptual estimation	0.22	0.25
Contextual estimation	0.36	0.42*
Written comparison	0.38	0.30
Problem solving	0.58*	0.71†
Total score	0.63†*	0.72†
Score A	0.62†*	0.74†

(*) Moderate correlations ($0.40<r<0.59$); (†) Strong correlation ($0.60<r<0.79$); $p<0.05$. *Legend.* Zareki-R: Neuropsychological test battery for Number Processing and Calculation in Children, Revised; WISC-III: Wechsler Intelligence Scale for Children; SAT: School Achievement test.
